# Hyperexpression of HOXC13, located in the 12q13 chromosomal region, in well-differentiated and dedifferentiated human liposarcomas

**DOI:** 10.3892/or.2013.2760

**Published:** 2013-10-01

**Authors:** MONICA CANTILE, FRANCESCA GALLETTA, RENATO FRANCO, GABRIELLA AQUINO, GIOSUÈ SCOGNAMIGLIO, LAURA MARRA, MARGHERITA CERRONE, GABRIELLA MALZONE, ANGELA MANNA, GAETANO APICE, FLAVIO FAZIOLI, GERARDO BOTTI, ANNAROSARIA DE CHIARA

**Affiliations:** 1 Division of Pathology, Istituto Nazionale Tumori ‘Fondazione G. Pascale’-IRCCS, 80131 Naples, Italy; 2 Division of Muscolo-Skeletal Oncology, Istituto Nazionale Tumori ‘Fondazione G. Pascale’-IRCCS, 80131 Naples, Italy; 3 Division of Muscolo-Skeletal Surgery, Istituto Nazionale Tumori ‘Fondazione G. Pascale’-IRCCS, 80131 Naples, Italy

**Keywords:** HOX genes, chromosome 12q13-15, liposarcoma cells

## Abstract

Liposarcoma (LPS) is the most common soft tissue neoplasm in adults and is characterized by neoplastic adipocyte proliferation. Some subtypes of LPSs show aberrations involving the chromosome 12. The most frequent are t(12;16) (q13;p11) present in more than 90% of myxoid LPSs and 12q13-15 amplification in well-differentiated and dedifferentiated LPSs. In this region, there are important oncogenes such as CHOP (DDIT3), GLI, MDM2, CDK4, SAS, HMGA2, but also the HOXC locus, involved in development and tumor progression. In this study, we evaluated the expression of HOXC13, included in this chromosomal region, in a series of adipocytic tumors. We included 18 well-differentiated, 4 dedifferentiated, 11 myxoid and 6 pleomorphic LPSs as well as 13 lipomas in a tissue microarray. We evaluated the HOXC13 protein and gene expression by immunohistochemistry and quantitative PCR. Amplification/translocation of the 12q13-15 region was verified by FISH. Immunohistochemical HOXC13 overexpression was observed in all well-differentiated and dedifferentiated LPSs, all characterized by the chromosome 12q13-15 amplification, and confirmed by quantitative PCR analysis. In conclusion, our data show a deregulation of the HOXC13 marker in well-differentiated and dedifferentiated LPSs, possibly related to 12q13-15 chromosomal amplification.

## Introduction

Liposarcoma (LPS) is one the most common sarcomas in adulthood. It is divided into different histotypes with different biological characteristics and clinical behavior. Thus, the correct classification is required for the prognostic stratification of patients and proper therapeutic approach. Well-differentiated LPSs (WDLPSs) (40–45% of all LPSs) tend to recur locally but do not metastasize, while the myxoid LPSs (MLPSs), if associated with higher hypercellularity, have a poor prognosis (1,2). LPSs are associated with a variety of molecular and genetic alterations that focus primarily on the short arm of chromosome 12 (3).

These alterations include translocations t(12;16) (q13;p11) and t(12;22) (q13;q12), which were essentially found in MLPS, and amplification of chromosomal region 12q13-15, associated with atypical lipomatous tumors\WDLPSs and dedifferentiated LPSs (DDLPS) (2,3).

The chromosomal region 12q13-15 contains ~164 genes. As shown by immunohistochemistry and quantitative RT-PCR analyses, some of these genes are systematically overexpressed in WDLPSs and DDLPSs. In particular, MDM2 (12q15) and CDK4 (12q14.1) are consistently amplified and overexpressed in WDLPS/DDLPS (4,7).

In this region, there are also other genes whose function has been associated with carcinogenesis, including CHOP (DDIT3), SAS and HMGA2, frequently rearranged/overexpressed in human sarcomas (3,5–7), an entire cluster of basic cytokeratins and the HOX C locus genes (8).

Homeobox genes are transcription factors that function during normal development (9) and contain the homeobox, a 183-bp DNA sequence coding for a 61-amino acid homeodomain. In mice (hox genes) and humans (HOX genes) there are 39 genes organized into four genomic clusters of ~100 kb in length, defined as HOX loci, each localized on a different chromosome (HOX A at 7p15.3, HOX B at 17p21.3, HOX C at 12q13.3 and HOX D at 2q31) (10).

Numerous studies associate abnormal expression of HOX genes to the development of various types of human cancer (11–21). In particular, several genes of the HOX C locus, are frequently overexpressed in several neoplasia (22–27).

Preliminary data, carried out on a multitumor tissue array to investigate HOXC13 distribution on several types of human cancer (unpublished data), showed the aberrant expression of HOXC13 protein in a small series of LPSs. In the present study, we evaluated HOXC13 expression in a whole spectrum of adipocytic tumors, including lipomas, WDLPSs, DDLPSs, pleomorphic (PLPS) and MLPSs, associating this analysis to the evaluation of amplification/translocation status of chromosomal region 12q13-15.

## Materials and methods

### Patients and specimens

Histological blocks and fresh cryostored tissues of 57 patients with WDLPSs, DDLPSs, MLPSs, PLPSs and lipomas, were selected from the files of the Pathology Unit of the National Cancer Institute Fondazione ‘G. Pascale’ of Naples. All patients were Caucasians and all provided written informed consent according to the institutional regulations.

This study was approved by the Ethics Committee of the National Cancer Institute ‘G. Pascale’. All diagnoses were established according to the World Health Organization Classification of Tumors (28).

Medical records were reviewed for clinical information. In addition, all cases were reviewed by expert pathologists (A.D.C. and G.B.), in order to confirm the diagnosis.

### TMA building

Fifty-seven tissue samples were used for a tissue microarray (TMA) building, using the most representative areas from each single case. Discrepancies between two pathologists for the same case were resolved with a joint analysis. Tissue cylinders with a diameter of 0.6 mm were punched from morphologically representative tissue areas of each donor tissue block and brought into one recipient paraffin block (3×2.5 cm) using a semiautomatic tissue arrayer (Galileo TMA).

### Immunohistochemistry

Immunohistochemical staining was carried out on TMA slides to evaluate the expression of HOXC13 marker. Paraffin slides were then deparaffinized in xylene and rehydrated through graded alcohols. Antigen retrieval was performed by microwave pretreatment in 0.01 M citrate buffer for 10 min. After protein block (BSA 5% in 1X PBS), the slides were incubated with primary antibody to human HOXC13 (cod. ab55251, dilution 1:1,200; Abcam, Cambridge, UK) overnight. Sections were incubated with mouse anti-rabbit or goat anti-mouse secondary IgG biotinylated secondary antibody for 30 min. Immunoreactivity was visualized by means of avidin-biotin-peroxydase complex kit reagents (Novocastra, Newcastle, UK) as the chromogenic substrate. Finally, sections were weakly counterstained with hematoxylin and mounted. Human hair follicles were used as positive controls. Irrelevant rabbit or mouse IgG antibodies were applied to negative control. Results were interpreted using a light microscope by 2 investigators (R.F. and A.D.C.).

For HOXC13, cytoplasmic and membrane staining were considered. Tissues were scored semi-quantitatively by evaluating the proportion of positive tumor cells over the total number of tumor cells (percentage of positive tumor cells per tissue microarray punch). Negative (score 0), low expression cases, and high expression cases were recorded when neoplastic cells expressing HOXC13 were comprised between 0 and 10% (score 1+), <30% (score 2+) and >30% (score 3+), respectively.

### RNA extraction from fresh and paraffin-embedded tissues

The sections obtained from paraffin-embedded samples were incubated at 37°C in the presence of xylene for ~20 min. Total RNA was purified using High Pure FFPE RNA Micro kit (Roche) following the manufacturer’s instructions. Total RNA was isolated from fresh tissues, using RNeasy Mini kit (Qiagen GmbH, Hilden, Germany) following the manufacturer’s instructions. All samples were treated with RNase-free DNase (Qiagen GmbH) to prevent amplification of genomic DNA. A total of 1 μg RNA was subjected to cDNA synthesis for 1 h at 37°C using the Ready-To-Go You-Prime First-Strand Beads kit (cod. 27-9264-01; Amersham Biosciences Europe Gmbh, Freiburg, Germany) in a reaction mixture containing 0.5 μg random hexamers (GeneAmp RNA PCR Random Hexamers Set N808-0127; Applied Biosystems, Foster City, CA, USA).

### Real-time PCR

Quantitative RT-PCR was performed in a LightCycler system (Roche Molecular Biochemicals, Mannheim, Germany) using TaqMan^®^ analysis. All reactions were run in glass capillaries with the LightCycler TaqMan Master Mix (cod. 04735536001; Roche Molecular Biochemicals), 10 μl, in a volume of 20 μl containing 2 μl of cDNA and 1 μl of specific TaqMan Gene Expression Assays for human HOXC13 (Real-Time Designer Assay cod. 04162498001; Roche Molecular Biochemicals), according to the manufacturer’s instructions. All reactions were performed in triplicate. The thermal cycling conditions included a step of 20 sec at 95°C followed by 40 cycles of 95°C for 1 sec and 60°C for 20 sec. The comparative C_t_ method was employed to determine the human HOXC13 gene variation, using as reference gene TaqMan Endogenous Controls Human ACTB (β-actin) Endogenous Control (Real-Time Designer Assay cod. 05532957001; Roche Molecular Biochemicals). We identified a calibrator cell line that represents the unitary amount of the target of interest and, consequently, the samples express n-fold mRNA relative to the calibrator. Final amounts of target were determined as follows: Target amount = 2-Ct, where Ct = [Ct (HOXC13) − Ct (ACTB)]_sample_ − [Ct (HOXC13) − Ct (ACTB)]_calibrator_.

### FISH analysis

TMA paraffin block sections cut at 4 μm were mounted on Superfrost/Plus microscope slides (Fisher Scientific, Pittsburgh, PA, USA). Slides were deparaffinized in xylene, dehydrated in 100% ethanol and then allowed to dry. They were placed in pre-treatment solution (Vysis) at 80°C for 10 min, followed by a rinse in purified water for 3 min. The slides were digested at 37°C for 15 min in 62.5 ml of 0.2 N HCl containing 250 mg protease (2,500–3,000 U/mg; Vysis) and rinsed in purified water for 3 min. The slides were then dehydrated through a series of graded ethanol solutions for 1 min each and allowed to air dry. For the cytogenetic investigation, we used the LSI *CHOP* Dual Color Break Apart Rearrangement Probe that contains a Spectrum Orange-labeled probe that spans a 700-kb region just centromeric of the *CHOP* (*DDIT3*) gene, and a Spectrum Green-labeled probe that spans a 660-kb region just telomeric of the *CHOP* (*DDIT3*) gene. Hybridization was performed by placing the slides in a humidified chamber at 37°C for overnight incubation. Following hybridization, rubber cement and coverslips were removed. Slides were treated in a posthybridization wash of 2X SSC containing 0.3% Nonidet P-40 at 73°C for 2 min and then transferred to ambient temperature 2X SSC/0.3% Nonidet P-40 for 5–60 sec. Slides were air dried and coverslip mounted with 4′-6-diamidino-2-phenylindole (DAPI, Vector Laboratories, Burlingame, CA, USA) nuclear counterstain. The sections were viewed using an Olympus BX41 (Melville, NY, USA) fluorescent microscope with a dual orange/green filter and were interpreted by 2 investigators (R.F. and G.A.).

The presence of 2 fusion signals/nucleus indicated an intact *CHOP* (*DDIT3*) gene. The presence of a single orange and single green signal indicated a rearranged *CHOP* (*DDIT3*) gene. Break apart with translocation of the *CHOP* (*DDIT3*) gene was observed in all evaluable MLPSs.

### Statistical analysis

The association between HOXC13 expression with other clinicopathological parameters was conducted using the χ^2^ and Student’s t-test.

Pearson’s χ^2^ test was used to determine whether a relationship exists between the variables included in the study. P<0.05 was considered to indicate a statistically significant difference. All statistical analyses were carried out using the Statistical Package for Social Sciences 8.0 software (SPSS Inc., Chicago, IL, USA).

## Results

### Clinicopathological characteristics of LPS tumors

The main clinical and pathological data are reported in Table I. In our histological samples, there were 18 WDLPSs, 9 DDLPSs, 11 MLPSs, 6 PLPSs and 13 lipomas. Twenty five of the 57 patients (43%) were female. The age of the patients ranged from 17 to 91 years, with an average of 57 years. Three samples are represented by recurrences from the same patients. Of the 57 samples, 12 (21%) were retroperitoneal, 33 (57%) were thigh location, 2 (3%) were shoulder location and 2 (3%) were subclavicular location, while of the remaining samples, one was hypogastric region, one gluteus, one forearm, one arm, one dorsum hand, one dorsum foot, one vulva region and one neck location.

### Expression of HOXC13 protein in LPS tissue microarray

**Figure 1:**
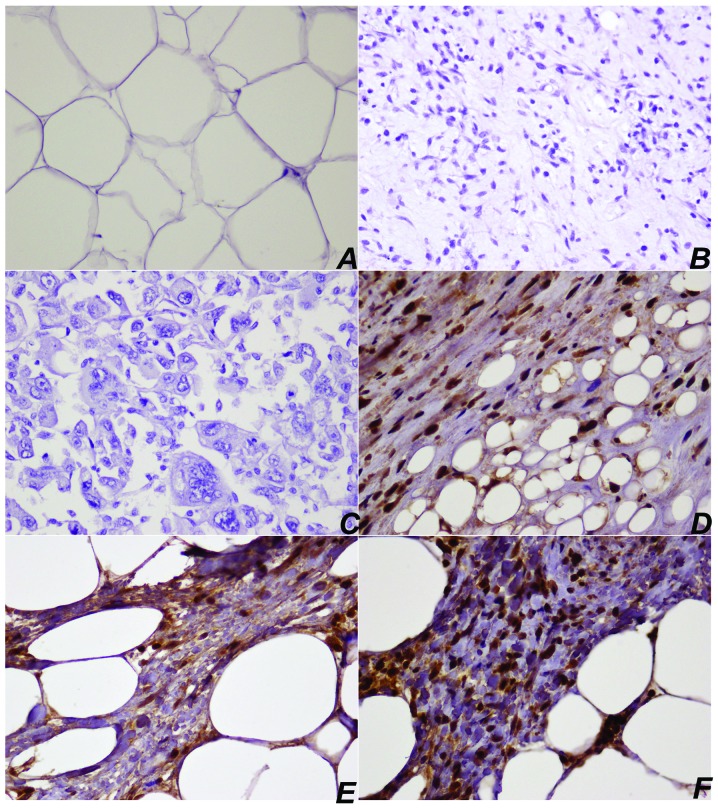
HOXC13 immunostaining in liposarcoma (LPS) tissues. (A) HOXC13 immunonegativity in lipoma sample (×40); (B) HOXC13 immunonegativity in myxoid LPS sample (×40); (C) HOXC13 immunonegativity in pleomorphic LPS sample (×40); (D) Moderate HOXC13 immunopositivity in dedifferentiated LPS sample (score 2+) (×40); (E) Moderate HOXC13 immunopositivity in well-differentiated (WD) LPS sample (score 2+) (×40); (F) High HOXC13 immunopositivity in WDLPS sample (score 3+) (×40).

Immunohistochemical detection of HOXC13 protein in 11/18 (61%) WDLPSs was scored as 3+, in 5/18 (27%) WDLPSs as 2+, while in only 1 case as 1+. In DDLPSs, HOXC13 was scored as 3+ in 4/9 (44%) samples, 2+ in 3/9 (33%) samples and 1+ in 2/9 (22%) tissues. In these cases, nuclear expression was observed in the nucleus of both neoplastic adipocyte-like cells and lipoblast. In MLPSs, there was only 1 sample with score 1+, while in PLPSs, 2 samples scored 1+. In all lipoma HOXC13 samples expression was absent (Fig. 1, Table I).

### HOXC13 mRNA quantification in LPSs

HOXC13 gene expression was evaluated in 20 selected fresh and paraffin-embedded tissue samples by real-time PCR quantification.

**Figure 2:**
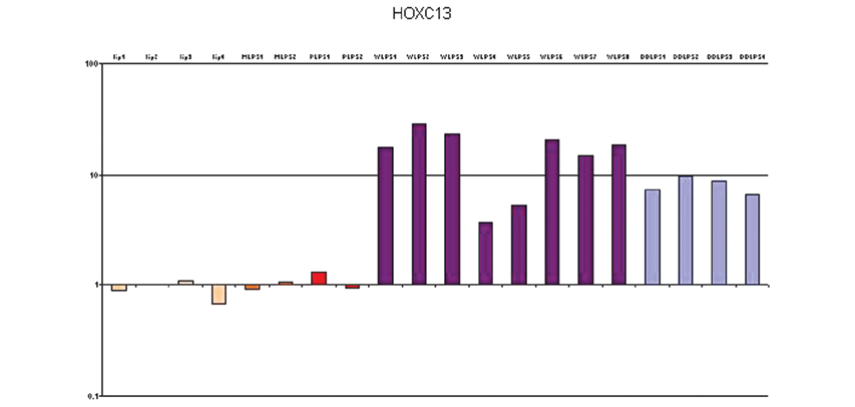
HOXC13 real-time expression in lipoma (lip), myxoid (MLPS), pleomorphic (PLPS), well-differentiated (WDLPS) and dedifferentiated (DDLPS) liposarcoma (LPS) tissues. All reactions were performed in triplicate and data are expressed as means of relative amount of mRNAs levels.

In 4 lipoma samples, in 2 MLPSs and in 1 PLPS, HOXC13 gene expression was absent, while it was very low in the other PLPS. Moreover, in 6/8 WDLPSs, a significant increase in HOXC13 mRNA expression (between 10 and 100-fold increase) was observed, while in the other 2 cases, the increase of expression was moderate (between 8 and 10-fold). In all 4 DDLPSs, there was a moderate increase of expression (~10-fold) (Fig. 2).

### Cytogenetic analysis

**Figure 3:**
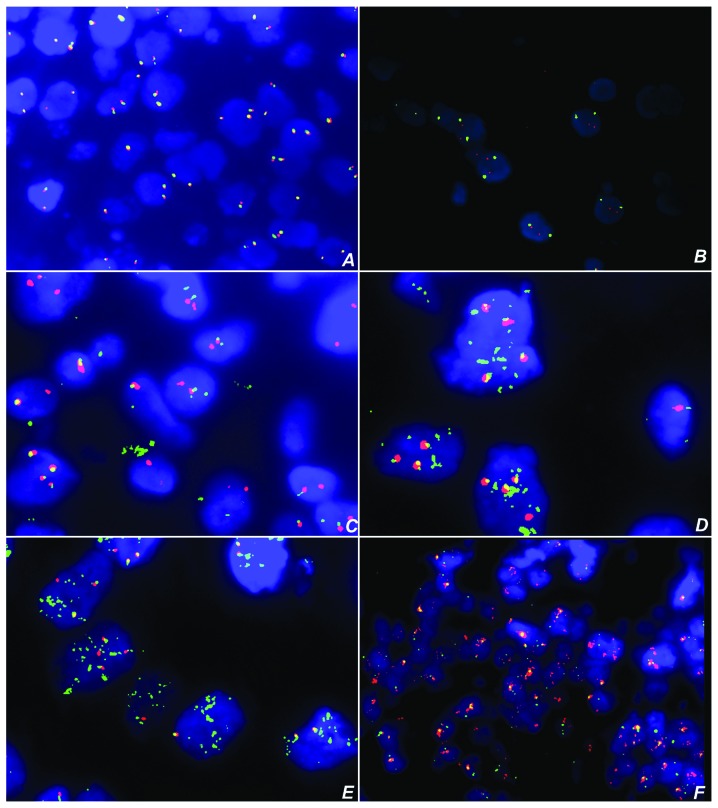
LSI *CHOP* Break Apart Rearrangement in liposarcoma (LPS) tissues. (A) Two fusion signals in lipoma (not rearranged gene); (B) two green and orange signals in myxoid LPS (rearranged gene); (C) two fusion signal in pleomorphic LPS (not rearranged gene); (D) two orange signals and increased green signals in dedifferentiated LPS (green copy gain without rearranged gene); (E) two orange signals and increased green signals in well-differentiated LPS (green copy gain without rearranged gene); (F) increase of both the green and orange signals in well-differentiated LPS (amplification signal without rearranged gene).

Break apart with translocation of the *CHOP* (*DDIT3*) gene was seen in all evaluable MLPSs (Fig. 3, Table I). Amplification of 660-kb green fluorophore-labelled probe was seen in 13/18 (72%) WDLPSs, while 660-kb green and 700-kb orange amplifications were present in 5/18 (27%) WDLPSs. Amplification of 660-kb green fluorophore-labelled probe was seen in 6/9 (66%) samples of DDLPS, and 660-kb green and 700-kb orange amplifications were present in 2/9 (22%) DDLPS. In only 1 case of PLPS, 660-kb green and 700-kb orange amplifications were present, while the remaining LPSs and lipomas showed no evidence of translocation and amplification of 660-kb or 700-kb probe (Fig. 3, Table I).

### Statistical investigations

Square analyses (χ^2^) showed no significant association between HOXC13 expression and clinical characteristics of LPS patients (Table II).

HOXC13 overexpression was strongly associated with WDLPS and DDLPS histotypes (P-value <0.001) and with amplification of chromosomal area detected by FISH probe (P-value <0.001) (Table II).

## Discussion

Liposarcoma (LPS) is the most common neoplasm of soft tissues and, although it rarely metastasizes, this tumor may reach considerable size, infiltrating adjacent anatomical structures. Similar to other sarcomas, this tumor is genetically characterized by a series of well-studied and highly specific chromosomal alterations (1–3).

The identification of these cytogenetic abnormalities, along with the morphological characterization, has assumed an increasingly important role, not only for a correct diagnostic definition, but also for prognostic stratification of patients, with marked therapeutic implications (2).

Most of the molecular abnormalities that characterize some tumors, including LPSs, involve the short arm of chromosome 12, in particular the q13–15 region. This region, in addition to the known oncogenes CHOP, MDM2, CDK4, SAS, GLI, HMGA2, also co-localizes an entire gene locus, HOX C, belonging to the HOX genes network (29–36); it includes 9 genes that were thoroughly studied in the evolution and neoplastic progression of various human organs and tissues (22–27). Furthermore, in other types of human cancer, such as bladder cancer, the amplification of this chromosomal area was associated not only with amplification of these genes, but also with overexpression of some genes of the HOX C locus (25).

Preliminary results, obtained by immunohistochemistry on a Multi-Tumor Array, in which a large spectrum of human cancer types was included, showed an increased HOXC13 expression particularly in LPS samples (data not shown).

Based on these data, in the current study, we analyzed a series of adipocytic tumors, including well-differentiated LPSs (WDLPSs), dedifferentiated LPSs (DDLPS), myxoid LPSs (MLPSs), pleomorphic LPSs (PLPSs) and lipomas, in order to evaluate HOXC13 expression and 12q13-15 chromosomal locus status.

Immunohistochemical analyses showed HOXC13 overexpression in most WDLPSs and DDLPSs. In addition, the data were confirmed by real-time PCR analysis, and are higher in WDLPSs and DDLPSs compared to other histological subtypes and lipomas.

The entire HOX C locus is localized in the same chromosomal region detected by LSI *CHOP* Dual Color Break Apart Rearrangement Probe. All samples of WDLPSs and DDLPS always show a higher number of green signals and, in some cases, of both orange and green signals.

It has been clearly demonstrated that the pathogenesis of LPSs could be directly connected to the block of adipocyte differentiation processes. In particular, it has been reported that the overexpression of CHOP protein in LPSs suppresses adipogenic conversion of preadipocytes through inhibition of C/EBP α gene expression (37). Moreover, the molecular mechanism underlying the activity of the anticancer drug trabectedin in LPS cells has been investigated. This molecule targeted selectively a specific FUS-CHOP chimeric transcript, promoting adipocyte differentiation, blocking the proliferation of neoplastic cells (38).

Numerous observations have linked genes regulating embryonal development to adipogenesis and lipidic metabolism (39). The HOX gene network plays a primary role in transcriptional regulation of human adipogenesis. Thus, these genes show a highly marked expression in adipose tissue and, moreover, their expression appears to vary in the different bodily deposits of white and brown adipose tissue (40). Therefore, there may be a role of HOX genes in the evolution of neoplastic tumors linked to the processes of adipocyte differentiation.

Based on our data, we hypothesized that the overexpression of HOXC13 in WDLPS and DDLPSs, with amplification of 12q13-15 region, may be involved in the pathogenesis of these tumors.

Since the amplification of the 12q13-15 region appears to be present in almost all WDLPSs and DDLPSs, identification of all genes within this area, which are altered in their expression and thus directly implicated in the pathogenesis of LPSs, represents an important aim of the clinic research for this malignancy. Moreover, the specific expression in WDLPS compared to lipomas may also be a significant tool for differential diagnosis between these two entities with overlapping characteristics.

The possibility of modifying, with a high efficiency, the expression and consequently the activity of HOX genes strictly associated with tumor development has previously been reported (41–44). Therefore, the possibility of interfering with HOXC13 gene expression could provide significant insight into a better understanding of the pathogenesis of this disease, and may aid in identifying new potential therapeutic targets.

## Figures and Tables

**Table I:** Clinicopathological characteristics of liposarcoma patients and tumors with respect to 12q13-15 chromosomal rearrangement and HOXC13 expression.

Case no.	Gender/Age	Histological subtype	Primary (P)/Recurrence (R)	Location	Chr.12q13-15 rearrangement	HOXC13 score
1	M/44	WDLPS	P	Left thigh	Green ampl	3+
2	F/66	WDLPS	P	Right thigh	Green ampl	3+
3	M/65	WDLPS	P	Retroperitoneal	Green/orange ampl	3+
4	F/35	WDLPS	P	Right thigh	Green ampl.	2+
5	M/49	WDLPS	P	Right thigh	Green/orange ampl	Not evaluable
6	M/61	WDLPS	P	Retroperitoneal	Green ampl	3+
7a	M/82	WDLPS	P	Left thigh	Green ampl	2+
7b	M/82	WDLPS	R	Left thigh	Green ampl	2+
8	M/75	WDLPS	P	Retroperitoneal	Green ampl	2+
9	M/39	WDLPS	P	Left thigh	Green/orange ampl	3+
10a	M/91	WDLPS	P	Left thigh	Green ampl	3+
10b	M/91	WDLPS	R	Left thigh	Green/orange ampl	1+
11	M/43	WDLPS	P	Left thigh	Green/orange ampl	3+
12	F/39	WDLPS	P	Right thigh	Green ampl	2+
13	M/69	WDLPS	P	Right thigh	Green ampl	3+
14	F/46	WDLPS	P	Left thigh	Green ampl	3+
15	F/41	WDLPS	P	Left thigh	Green ampl	3+
16	F/65	WDLPS	P	Retroperitoneal	Green ampl	3+
17	F/65	DDLPS	P	Retroperitoneal	Not evaluable	1+
18	M/75	DDLPS	P	Retroperitoneal	Green/orange ampl	2+
19	M/62	DDLPS	P	Retroperitoneal	Green/orange ampl	3+
20	M/53	DDLPS	P	Retroperitoneal	Green ampl	1+
21	F/90	DDLPS	P	Left thigh	Green ampl	3+
22a	F/78	DDLPS	P	Retroperitoneal	Green ampl	3+
22b	F/78	DDLPS	R	Retroperitoneal	Green ampl	2+
23	M/60	DDLPS	P	Retroperitoneal	Green ampl	2+
24	M/69	DDLPS	P	Hypogastric region	Green ampl	3+
25	F/51	MLPS	P	Right thigh	Translocation	0
26	M/39	MLPS	P	Left thigh	Translocation	0
27	F/37	MLPS	P	Right thigh	Translocation	0
28	M/66	MLPS	P	Right thigh	Translocation	1+
29	M/48	MLPS	P	Left thigh	Translocation	0
30	F/42	MLPS	P	Right thigh	Translocation	0
31	F/51	MLPS	P	Right thigh	Translocation	0
32	M/34	MLPS	P	Right thigh	Translocation	0
33	M/62	MLPS	P	Left gluteus	Translocation	0
34	F/49	MLPS	P	Right thigh	Translocation	0
35	F/43	MLPS	P	Left thigh	Translocation	0
36	M/61	PLPS	P	Right thigh	Green/orange ampl	1+
37	M/62	PLPS	P	Left thigh	No	0
38	M/48	PLPS	P	Right thigh	No	0
39	F/52	PLPS	P	Left forearm	No	1+
40	M/51	PLPS	P	Retroperitoneal	No	0
41	M/76	PLPS	P	Left thigh	No	0
42	F/57	Lipoma		Left shoulder	No	0
43	M/45	Lipoma		Right thigh	Not evaluable	0
44	F/66	Lipoma		Right arm	No	0
45	M/62	Lipoma		Left thigh	No	0
46	F/60	Lipoma		Left dorsum hand	No	0
47	M/47	Lipoma		Right subclavicular	No	0
48	F/46	Lipoma		Left subclavicular	No	0
49	F/60	Lipoma		Left vulva	No	0
50	M/66	Lipoma		Neck	No	0
51	F/17	Lipoma		Left thigh	No	0
52	F/50	Lipoma		Right shoulder	No	0
53	M/33	Lipoma		Left thigh	No	0
54	F/66	Lipoma		Right dorsum foot	No	0

M, male; F, female; WDLPS, well-differentiated liposarcoma; DDLPS, dedifferentiated liposarcoma; MLPS, myxoid liposarcoma; PLPS, pleomorphic liposarcoma.

**Table II:** Relationship between HOXC13 protein expression and clinical characteristics, histological subtypes and chromosomal 12q13-15 rearrangement in liposarcoma patients.

	HOXC13 score, n (%)					
			
Characteristic	0	1	2	3	Total, n	P-value
Gender/Age (years)						
Female	14 (58.33)	2 (8.33)	2 (8.33)	6 (25)	24	0.812
Male	13 (44.83)	3 (10.34)	4 (13.79)	9 (31.03)	29	
Age (years; mean, 57)						
≤57	18 (66.67)	2 (7.41)	2 (7.41)	5 (18.52)	27	0.142
>58	9 (64.62)	3 (11.54)	4 (15.38)	10 (38.46)	26	
Histological subtype						
Lipoma	13 (100)	0	0	0	13	<0.001
WDLPS	0	0	4 (26.67)	11 (73.33)	15	
DDLPS	0	2 (25)	2 (25)	4 (50)	8	
MLPS	10 (90.91)	1 (9.09)	0	0	11	
PLPS	4 (66.67)	2 (33.33)	0	0	6	
12q13-15 rearrangement						
Green ampl	0	2 (8.69)	6 (26.09)	15 (65.22)	17	<0.001
Translocation	10 (90.91)	1 (9.09)	0	0	11	
No rearrangement	16 (94.12)	1 (5.88)	0	0	17	

WDLPS, well-differentiated liposarcoma; DDLPS, dedifferentiated liposarcoma; MLPS, myxoid liposarcoma; PLPS, pleomorphic liposarcoma.
